# Increased serum concentration of ceramides in obese children with nonalcoholic fatty liver disease

**DOI:** 10.1186/s12944-018-0855-9

**Published:** 2018-09-12

**Authors:** Natalia Wasilewska, Anna Bobrus-Chociej, Ewa Harasim-Symbor, Eugeniusz Tarasów, Małgorzata Wojtkowska, Adrian Chabowski, Dariusz M. Lebensztejn

**Affiliations:** 1 Department of Pediatrics, Gastroenterology, Hepatology, Nutrition and Allergology, Medical University of Bialystok, ul. Waszyngtona 17, 15-274 Białystok, Poland; 2 Department of Physiology, Medical University of Bialystok, Bialystok, Poland; 3 Department of Radiology, Medical University of Bialystok, Bialystok, Poland; 4 Department of Radiology, University Teaching Children’s Hospital, Bialystok, Poland

**Keywords:** Obesity, NAFLD, Ceramides, Fatty liver, Magnetic resonance proton spectroscopy

## Abstract

**Background:**

Hepatic lipid accumulation is closely related to the development of insulin resistance, which is regarded as one of the most significant risk factors of nonalcoholic fatty liver disease (NAFLD). Although the exact molecular pathway leading to impaired insulin signaling has not been definitively established, ceramides are suspected mediators of lipid induced hepatic insulin resistance. Therefore, the aim of the study was to evaluate the serum ceramides concentration in obese children with NAFLD.

**Methods:**

The prospective study included 80 obese children (aged 7–17 years, median 12 years) admitted to our Department to diagnose initially suspected liver disease. Patients with viral hepatitis (HCV, HBV, CMV), autoimmune (AIH), toxic and metabolic (Wilson’s disease, alfa-1–antitrypsin deficiency) liver diseases and celiac disease were excluded. NAFLD was diagnosed based on pediatric diagnostic criteria in obese children with liver steatosis in ultrasound (US) as well as elevated alanine transaminase (ALT) serum activity after exclusion of other major liver diseases listed before. Ultrasonography was used as a screening method and for qualitative assessment of the steatosis degree (graded according to Saverymuttu scale). Advanced steatosis was defined as a score > 1. The total intrahepatic lipid content (TILC) was assessed by magnetic resonance proton spectroscopy (^1^HMRS) which is the most accurate technique for assessment of ectopic fat accumulation. Fasting serum concentration of ceramides was measured in 62 children.

**Results:**

NAFLD was diagnosed in 31 children. Significant, positive correlation was found between total serum concentration of ceramides and insulin (*r* = 0.3, *p* = 0.02) and HOMA-IR (*r* = 0.28, *p* = 0.03). Total ceramide concentration as well as specific fatty acid-ceramides (FA-ceramides) concentrations, namely: myristic, palmitic, palmitoleic, stearic, oleic, behenic and lignoceric were significantly higher (*p* = 0.004, *p* = 0.003, *p* = 0.007, *p* < 0.001, *p* = 0.035, *p* = 0.008, *p* = 0.003, *p* = 0.006, respectively) in children with NAFLD compared to controls (*n* = 14). Moreover, children with NAFLD had significantly higher activity of ALT (*p* < 0.001) and GGT (*p* < 0.001), HOMA-IR (*p* = 0.04), BMI (*p* = 0.046), waist circumference (*p* = 0.01) steatosis grade in ultrasound (*p* < 0.001) and TILC in ^1^HMRS (*p* < 0.001) compared to children without NAFLD. We did not find significant differences in total and FA-ceramide species concentrations between children with mild (grade 1) and advanced liver steatosis in ultrasonography (grade 2–3).

**Conclusion:**

Elevated ceramide concentrations in obese patients together with their significant correlation with insulin resistance parameters suggest their association with molecular pathways involved in insulin signaling impairment known to be strongly linked to pathogenesis of non-alcoholic fatty liver disease.

## Background

Childhood obesity caused mostly by sedentary lifestyle and hypercaloric diet has become a major worldwide health problem [1]. The global prevalence of obesity among children aged 5–19 is estimated to be 5.6% in girls and 7.8% in boys [2]. In Poland, the prevalence of overweight and obesity among children aged 7–18 years is reported to be over 14% in girls and over 18% in boys [3]. Following the current epidemic of obesity, the incidence of nonalcoholic fatty liver disease (NAFLD) has risen as well, making it a most common liver pathology worldwide. [4–6]. Certain features of metabolic syndrome such as visceral obesity, type 2 diabetes, hypertension and cardiovascular disease are the most common comorbidities of NAFLD. However NAFLD connection to metabolic syndrome is complex and bi-directional – the cause-and-effect relationship between them is still at the debate [7–9]. The term NAFLD has been applied to different degrees of liver steatosis – it includes changes from simple fat accumulation to steatohepatitis and fibrosis. NAFLD pathogenesis has not yet been fully established. A growing body of evidence suggest ‘multiple hits hypothesis’ based on assumption that lipid accumulation accompanied by insulin resistance increase susceptibility to liver damage by multiple factors such as lipotoxicity, adipocytokines, gut - derived endotoxin, endoplasmic reticulum stress and others [10]. Lipotoxicity, a process involving lipid induced oxidative stress, inflammation and cell death is among most commonly investigated mechanisms of liver injury. It has been recently established that not only the quantity but also quality of accumulated lipids plays a major role in NAFLD pathogenesis. A growing body of evidence suggest that it is the type of lipids, their relative amount and interplay that is of central importance in the process of lipotoxicity [11, 12]. Therefore the contribution of certain lipid species to NAFLD pathogenesis is commonly discussed. Although triglycerides represent a major class of accumulated lipids responsible for steatosis, they have been considered protective against cell injury [13]. Sphingolipids are another group of lipids that play significant role in cell homeostasis. They may serve as structural and signaling molecules or be transported to plasma lipoproteins. Many studies suggest their important role in metabolic syndrome. Altered sphingolipids metabolism has been found in obesity, type 2 diabetes, atherosclerosis and cardiovascular disease [14]. A recent review on the literature indicates that amongst sphingolipids ceramides (CER) may be the main lipid species involved in lipotoxicity during NAFLD [15, 16]. They have been also identified, alongside diacylglycerols as major mediator of lipid – induced insulin resistance and other cellular processes strongly linked to NAFLD – fat accumulation, oxidative stress, inflammation and cell death [17–20]. Some studies suggest that certain FA-ceramide species may be more pathogenic than the other ones. Ceramides containing long side chains such as palmitic (CER 16:0) and stearic (CER 18:0) are suspected to be major molecules involved in insulin resistance and hepatic steatosis [21, 22]. In recent years there has been considerable interest in the role of different lipid species in the pathogenesis of metabolic diseases however our knowledge on this subject in pediatric patients is based on limited data. Therefore the main aim of our research was to evaluate the serum ceramides concentration and their correlation with the steatosis degree, anthropometric measurements, insulin resistance and other biochemical parameters in obese children with NAFLD. The purpose of this analysis is to determine if ceramides are related to NAFLD pathogenesis and if they correlate with disease severity. Another objective of the study was to investigate if they may serve as a possible molecular mediator of lipid-induced hepatic insulin resistance.

## Methods

The prospective study included 80 children (60 males and 20 females) at median age 12 (7–17 years) with body mass index (BMI) > 95th percentile admitted to our department to diagnose initially suspected liver disease (elevated serum alanine aminotransferase (ALT) activity and/or ultrasonographic liver brightness and/or hepatomegaly). Patients with viral hepatitis (HCV, HBV, CMV), autoimmune (AIH), toxic (drug induced liver injury), selected metabolic liver diseases (Wilson’s disease, alfa-1–antitrypsin deficiency cystic fibrosis) and coeliac disease were excluded. Written informed consent was obtained from parents and all participants older than 16 years old. Reference group included 15 children with normal BMI (<85th percentile), without any somatic organ pathology at similar age and sex. The protocol was approved by the Bioethics Committee of the Medical University of Bialystok in accordance with the Declaration of Helsinki. All children underwent anthropometric measurements, their BMI was calculated and the corresponding percentile for their age and sex was established using OLAF growth charts [23]. The laboratory tests included activity of ALT, gamma glutamyltransferase (GGT), lipid profile (total cholesterol, lipoprotein-HDL and LDL, triglycerides) and insulin resistance indicators (glucose and insulin levels, calculated homeostasis model assessment-estimated insulin resistance (HOMA-IR) [24]). Ultrasonography was performed as a screening for liver steatosis in the beginning of the diagnostic process. The qualitative assessment of the degree of liver steatosis (graded according to Saverymuttu scale [25] was performed in a blinded fashion by ultrasonography performed by one radiologist. Advanced steatosis was defined as a score > 1. Nonalcoholic fatty liver disease was diagnosed based on criteria proposed by ESPGHAN Hepatology Commitee on diagnosing NAFLD in children. In pediatric clinical practice NAFLD diagnosis is suggested by finding liver steatosis in ultrasonoghraphy and elevated serum alanine transaminase activity in obese children with exclusion of other major causes of liver disease listed earlier as exclusion criteria [26]. The total intrahepatic lipid content (TILC) was assessed in relative units (r.u) in comparison to the unsuppressed water signal by magnetic resonance proton spectroscopy (^1^HMRS), which is acknowledged method for accurate evaluation of ectopic fat accumulation [27]. ^1^HMR spectroscopy was performed with a 1.5 T scanner (Picker Eclipse) and with PRESS sequencing. Fasting serum concentration of ceramides was measured in 62 children.

In order to quantify ceramides, a small volume of the chloroform phase containing lipids was carefully injected to a tube with N-palmitoyl-D-erythro-sphingosine (C17 base) as an internal standard. Thin-layer chromatography silica plates (Kieselgel 60, 0.22 mm, Merck, Darmstadt, Germany) with a heptane:isopropyl ether:acetic acid (60:40:3, vol/vol/vol) resolving solution were then used to separate lipid fractions. To visualize lipid bands, plates were sprayed with 0.2% solution of 3′7’ -dichlorofluorescin in methanol. Utraviolet light was then used to identify the bands using standards. The gel bands were removed from the plate, placed in the screw tubes and transmethylated with BF3/methanol. Next, HewlettPackard 5890 Series II gas chromatograph with Varian CPSIL capillary column (50 m 0.25 mm internal diameter) and flame-ionization detector (Agilent Technologies, Santa Clara, CA) was used to analyze fatty acid methyl esters (FAMEs) dissolved in hexane. The temperature of injector and detector was set at 250 C. The oven temperature was increased linearly from 160 to 225 C at a rate of 5 C/min. The long-chain fatty acids were quantified according to the retention times of standards. Total content of CER was estimated as the total of the particular fatty acid species of the assessed fraction and it was expressed in nanomoles per milliliter of the serum. Statistical analysis was performed using Statistica 12.0 software. The serum concentrations of biochemical parameters were expressed as median; 25–75 quartile (Q1-Q3). Mann-Whitney two-sample test was used for comparisons of nonparametric data. Correlations were analyzed by the Spearman rank-correlation test for non-parametric data and by the Pearson method for parametric data. The tests were considered statistically significant at *p* < 0.05.

## Results

The research included eighty obese patients (60 males and 20 females) as a study group and 15 children with normal BMI without any somatic organ pathology as a reference group. Median BMI was 27.79 (25.85–31.85) kg/m^2^. Nonalcoholic fatty liver disease was diagnosed in 31 children (38.75%). Mild steatosis was diagnosed in 49 (61.25%) and advanced in 31 (38.75%) children respectively. Insulin resistance was observed in 62 individuals (77.50%). Characteristics of the study group is presented in the Table 1.

**Table 1:** Characteristics of the study group (*n* = 80)

Parameter	Median (Q1-Q3
Age (years)	12 (11–15)
BMI (kg/m^2^)	27.8 (25.9–31.9)
Waist (cm)	94 (90–104)
ALT (IU/l)	35.5 (21.5–59.0)
GGT (IU/l)	21 (15.5–30.0)
Cholesterol (mg/dl)	177.5 (146.0–191.5)
HDL-cholesterol (mg/dl)	47.5 (39–54)
LDL – cholesterol (mg/dl)	99.5 (79.5–121.0)
Triglycerides (TG) (mg/dl)	107.5 (84.0–152.5)
Glucose (mg/dl)	92.0 (85.5–96.0)
Insulin (μIU/ml)	15.0 (11.7–19.9)
HOMA – IR	3.5 (2.6–4.5)
TILC (r.u)	107 (58–180)

Patients with the diagnosis of NAFLD had significantly higher waist circumference (*p* < 0.05), GGT activity (*p* < 0.001), HOMA-IR (*p* < 0.05) and liver lipid content in ^1^HMRS (*p* < 0.001) when compared to the rest of obese patients. Similar differences were observed in comparison between NAFLD patients and non-hepatopathic obese children (no steatosis or ALT elevation) (Table 2).

**Table 2:** Comparison of selected parameters between the group of NAFLD patients and non-hepatopathic obese patients

Parameter	NAFLD group (*n* = 31) Median (Q1-Q3)	Non – hepatopathic obese (*n* = 12) Median (Q1-Q3)	P
Age (years)	14 (11–16)	12 (10.5–14)	ns
BMI (kg/m^2^)	28.1 (26.6–33.5)	26.2 (25.3–29.5)	0.035
Waist (cm)	98 (94–107)	87 (82–94)	0.004
ALT (IU/l)	64 (51–104)	18 (15–21)	< 0.001
GGT (IU/l)	30 (22–45)	15.0 (13.0–18.5)	< 0.001
Cholesterol (mg/dl)	181 (159–216)	162.5 (139.5–182.0)	ns
HDL-cholesterol (mg/dl)	45 (40–51)	50.5 (41–55.5)	ns
LDL – cholesterol (mg/dl)	105 (80–145)	89.5 (74.0–105.5)	ns
Triglycerides (TG) (mg/dl)	140 (85–167)	87.5 (73.0–121.0)	0.037
Glucose (mg/dl)	93 (84–99)	91 (86–96)	ns
Insulin (μIU/ml)	17.4 (13.8–21.0)	14.8 (10.3–17.7)	ns
HOMA – IR	3.9 (2.8–4.8)	3.4 (2.3–3.8)	ns
TILC (r.u)	172 (120–216)	29 (17–58)	< 0.001

**Fig. 1:**
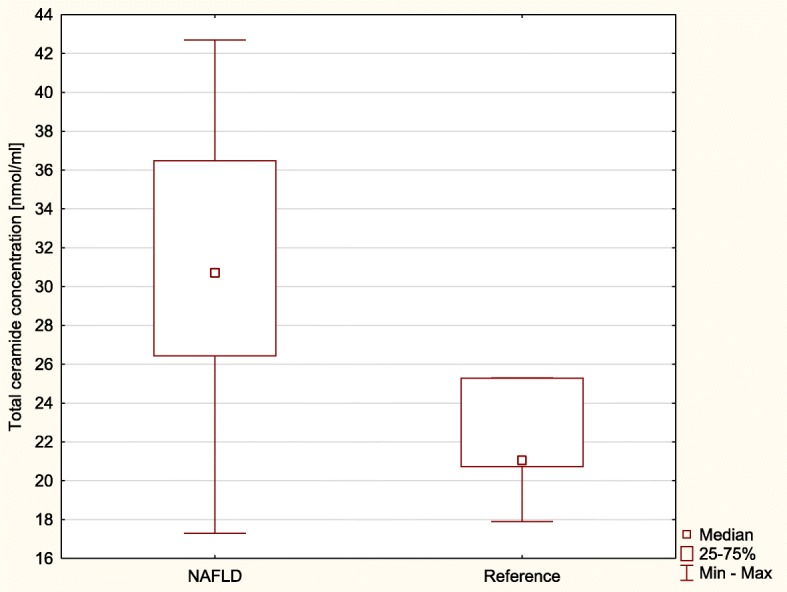
Total ceramide concentrations in serum of the patients with NAFLD (NAFLD) and reference group (Reference)

Total ceramide concentration was significantly higher in the study group than in the reference group (*p* < 0.001). Likewise, group of children with NAFLD reported significantly higher total ceramide concentration than the reference group (*p* = 0.004) (Fig. 1). Selected FA-ceramide concentrations assessed in the study group in comparison to reference group are presented in Table 3. Further analysis has shown that patients with NAFLD had significantly higher concentration of certain FA-ceramides when compared to reference group (Table 4).

**Table 3:** Differences between serum FA-ceramide (CER) (nmol/ml) concentrations in study group and reference group

Ceramide (FA)	Study group M (Q1-Q3)	Reference group M (Q1-Q3)	*p* value
CER myristic (C14:0)	2.1 (1.4–4.3)	0.9 (0.7–1.2)	< 0.001
CER palmitic (C16:0)	8.6 (6.7–10.4)	5.2 (4.8–6.8)	< 0.001
CER palmitoleic (C16:1)	1.0 (0.8–1.3)	0.6 (0.5–0.8)	< 0.001
CER stearic (C18:0)	5.6 (4.5–7.6)	4.0 (3.6–4.5)	< 0.005
CER oleic (C18:1)	2.8 (2.4–3.6)	2.1 (1.9–2.8)	< 0.005
CER linoleic (C18:2)	0.3 (0.0–1.0)	0.2 (0.0–0.7)	ns
CER arachidic (C20:0)	0.5 (0.4–0.6)	0.4 (0.4–0.5)	ns
CER linolenic (C18:3)	0.3 (0.2–0.3)	0.2 (0.2–0.3)	ns
CER behenic (C22:0)	1.5 (1.3–1.7)	1.3 (1.1–1.4)	< 0.01
CER arachidonic (C20:4)	0.5 (0.4–0.7)	0.5 (0.4–0.7)	ns
CER lignoceric (C24:0)	3.3 (2.8–4.1)	3.0 (2.6–3.3)	< 0.05
CER eicosapentaenoic (C20:5)	0.8 (0.7–0.9)	0.8 (0.7–0.9)	ns
CER nervinic (C24:1)	2.3 (2.0–2.6)	2.0 (1.9–2.2)	ns
CER docosahexaenoic (C22:6)	0.7 (0.6–0.9)	0.7 (0.6–0.8)	ns

**Table 4:** Differences between serum FA-ceramide (CER) (nmol/ml) concentrations in children with NAFLD and reference group

Ceramide (FA)	NAFLD group M (Q1-Q3)	Reference group M (Q1-Q3)	*p* value
CER myristic (C14:0)	1.9 (1.3–2.3)	0.9 (0.7–1.2)	< 0.005
CER palmitic (C16:0)	7.8 (6.5–10.0)	5.2 (4.8–6.8)	< 0.01
CER palmitoleic (C16:1)	0.9 (0.7–1.1)	0.6 (0.5–0.8)	< 0.001
CER stearic (C18:0)	5.2 (4.3–6.8)	4.0 (3.6–4.5)	< 0.05
CER oleic (C18:1)	2.9 (2.4–3.7)	2.1 (1.9–2.8)	< 0.05
CER linoleic (C18:2)	0.5 (0.1–1.1)	0.2 (0.0–0.7)	ns
CER arachidic (C20:0)	0.5 (0.4–0.6)	0.4 (0.4–0.5)	ns
CER linolenic (C18:3)	0.3 (0.2–0.4)	0.2 (0.2–0.3)	ns
CER behenic (C22:0)	1.6 (1.4–1.7)	1.3 (1.1–1.4)	< 0.005
CER arachidonic (C20:4)	0.6 (0.4–0.7)	0.5 (0.4–0.7)	ns
CER lignoceric (C24:0)	3.7 (3.0–4.1)	3.0 (2.6–3.3)	< 0.01
CER eicosapentaenoic (C20:5)	0.7 (0.7–0.9)	0.8 (0.7–0.9)	ns
CER nervinic (C24:1)	2.3 (2.0–2.5)	2.0 (1.9–2.2)	ns
CER docosahexaenoic (C22:6)	0.7 (0.6–0.8)	0.7 (0.6–0.8)	ns

**Fig. 2:**
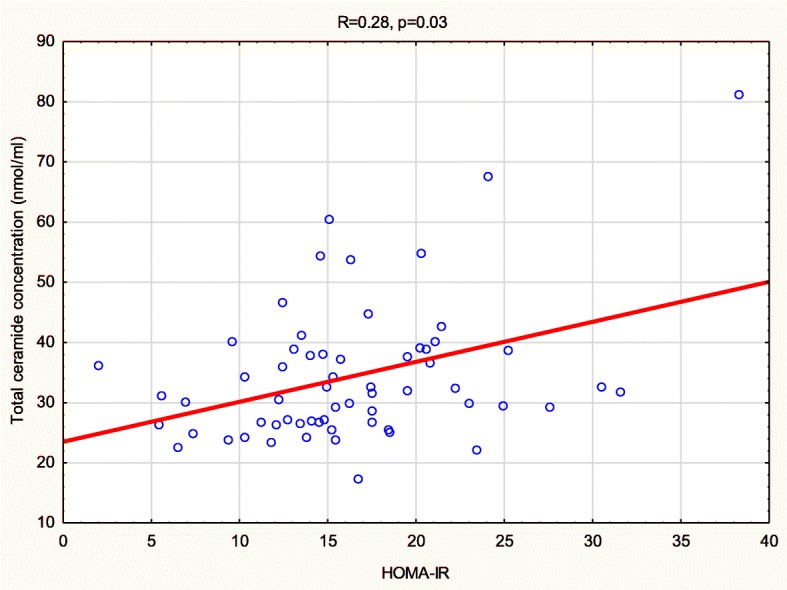
Correlation between serum total ceramide concentration and HOMA-IR index

Significant, positive correlation was found between serum total ceramide concentration and insulin concentration (*R* = 0.286) and HOMA-IR index (*R* = 0.278) (Fig. 2).

It did not correlate with transaminases, triglycerides, total cholesterol, HDL – cholesterol, LDL – cholesterol nor the amount of lipids measured in ^1^HMRS. Moreover CER lignoceric was positively correlated with HOMA-IR and insulin (*R* = 0.276, *R* = 265 respectively).

## Discussion

In the current research we assessed the concentration of circulating ceramides (total and FA-ceramide species) in obese pediatric patients in reference to their clinical and laboratory data. The results of this study show significantly higher total serum CER concentration in obese patients as well as patients with NAFLD when compared to controls. Moreover, we observed significantly higher levels of certain ceramides (C14:0, C16:0, C16:1, C18:0, C18:1, C22:0, C24:0) in the serum of obese children and patients with NAFLD in comparison to reference group. Another important finding was that total ceramide concentration was positively correlated with HOMA-IR and insulin levels.

In accordance with the present results, previous studies on humans and animals have demonstrated significantly increased total serum CER concentrations and specific FA – ceramides in obese populations [17, 18, 28]. However, among these studies, Haus et al. and Lopez et al. reported higher ceramide concentrations in obese populations with type 2 diabetes, disease known to be independently related to increased serum CER levels. On the other hand, study by Majumdar et al. did not report significant elevation of neither total serum CER nor specific CER in the group of overweight children in comparison to lean controls [29]. This discrepancy could be attributed to smaller study group and differences in inclusion criteria (overweight vs. obese). Previous studies on ceramide concentrations in NAFLD individuals performed on humans and mice report findings which support the results of our study. Significantly higher total CER concentration as well as a major increase in C16:0, C22:0, C24:0, C24:1 in NAFLD mice was noticed by Kasumov et al. [30]. Recent data suggest a major role of C16-ceramides overaccumulation in metabolic diseases, mainly due to impaired hepatic fatty acid oxidation. It has been hypothesized that long-chain FA-ceramides such as C16-ceramide increase liver susceptibility for diet-induced steatohepatitis and insulin resistance in obese individuals [21, 31]. Increased levels of C16:0 ceramide in serum of NAFLD patients seem to be consistent with these studies. Luukkonen et al. presented an elegant study on liver ceramide levels in adults with nonalcoholic fatty liver disease and provided an important evidence that ceramides may play a major role in triggering insulin resistance in individuals with NAFLD. In this study patients were divided into two groups – ‘metabolic NAFLD’ defined by insulin – resistance and PNPLA3 – associated NAFLD. The analysis revealed higher levels of C16:0, C18:0, C19:0, C20:0, C22:0, C24:1 dihydroceramides in the group of adults with ‘metabolic NAFLD’ [22]. This study points out up that composition of hepatic fatty acids lipidome impacts patient’s phenotype, unfortunately no correlation with plasma ceramides was done. Recent review on lipid accumulation in pediatric patients with NAFLD also strongly highlights the role of hepatic fat composition in progression of the disease and suggests some possible novel therapeutic targets [12]. Positive correlation between total ceramide concentration and insulin resistance parameters reported in this study is consistent with other research performed on adult and pediatric patients [17, 18, 22]. Taken together these studies provide important insights into the possible link of ceramides to pathogenesis of nonalcoholic fatty liver disease.

In the literature there are only few studies on serum ceramide concentration in pediatric population. This is a first study conducted in obese children with nonalcoholic fatty liver disease investigating serum ceramide concentration. The novelty of these findings is the main strength of our study. However there are some limitations to our study. We did not use histological examination in the diagnosis of NAFLD. According to ESPGHAN Hepatology Committee liver biopsy is required for definitive diagnosis of NAFLD, but is not proposed in screening [32]. This procedure is an ‘imperfect’ gold standard – it is invasive, there is a considerable variability of sample and it provides only static information. However it needs to performed in some unclear cases to distinguish NAFLD from other liver diseases. Other indications for liver biopsy include suspected advanced disease, young age, hepatosplenomegaly, considerable increase in ALT activity and before pharmacological intervention. Our patients did not meet these criteria, therefore liver biopsy was not performed [33]. Another limitation of our study is a use of indirect method of insulin resistance assessment (HOMA-IR), while the euglycemic hyperinsulinemic clamp technique is considered to be a golden standard for insulin sensitivity evaluation [34].

## Conclusion

To conclude, the purpose of the current study was to determine plasma ceramide concentration in obese adolescents. Elevated ceramide concentrations in obese patients together with their significant correlation with insulin resistance parameters suggest their association with molecular pathways involved in insulin signaling impairment known to be strongly linked to pathogenesis of non-alcoholic fatty liver disease. Further work is needed to determine exact role of ceramides in the pathogenesis of this disease but in the future it may serve as clinical tool and another potential therapeutic target.

## Abbreviations

^1^HMRS: Magnetic resonance proton spectroscopy
AIH: Autoimmune hepatitis
ALT: Alanine transaminase
BMI: Body mass index
CER: Ceramide
CMV: Cytomegalovirus
FA-Ceramide: Fatty acid – ceramide
GGT: Gamma glutamyltransferase
HBV: Hepatitis B virus
HCV: Hepatitis C virus
HDL: High-density lipoprotein
HOMA – IR: Homeostasis model assessment-estimated insulin resistance
LDL: Low-density lipoprotein
NAFLD: Nonalcoholic fatty liver disease
TILC: Total intrahepatic lipid content
US: Ultrasound
